# Differential T cell reactivation by two PD-L1 nanobodies through blockade alone or blockade with internalization

**DOI:** 10.1038/s41598-026-47884-x

**Published:** 2026-04-18

**Authors:** Ji Hyun Lee, Su Yeon Cho, Hee Eon Lee, Sukmook Lee

**Affiliations:** 1 Department of Biopharmaceutical Chemistry, Kookmin University, Seoul, 02707 Republic of Korea; 2 Department of Applied Chemistry, Kookmin University, Seoul, 02707 Republic of Korea; 3 Antibody Research Institute, Kookmin University, Seoul, 02707 Republic of Korea; 4 Department of Neurosurgery, School of Medicine, Stanford University, Palo Alto, CA 94304 USA

**Keywords:** Antigen depletion, Immune checkpoint blockade, Nanobody, PD-L1, Phage display, T cell activation, Cancer, Immunology

## Abstract

Programmed death-ligand 1 (PD-L1) plays a key role in tumor immune evasion by suppressing T cell activity through its interaction with programmed death-1 (PD-1). Although PD-L1 antibodies have significantly advanced cancer immunotherapy, clinical responses remain limited, associated with tumor heterogeneity, acquired resistance, and the structural and functional constraints of full-length immunoglobulin G formats. These limitations highlight the need for alternative PD-L1–targeting scaffolds that offer improved modularity and functional versatility. In this study, two PD-L1-specific nanobodies (variable domains of heavy-chain-only antibodies, VHHs; K113.1 and K113.2) were isolated using phage display and reformatted as Fc-fusion antibodies (VHH-Fc; K113.1-Fc and K113.2-Fc). Both VHH-Fc antibodies exhibited high-affinity binding to human PD-L1 (K_D_ < 3.4 nM) and effectively blocked PD-1/PD-L1 interactions. Notably, K113.2-Fc also induced PD-L1 internalization and surface downregulation, which was associated with enhanced T cell reactivation in vitro compared with blockade alone. Collectively, these findings indicate that closely related VHH-Fc antibodies can display distinct functional profiles, enabling direct comparison between blockade-only and blockade-plus-internalization mechanisms within the same scaffold. These VHH-based formats may provide useful tools for studying PD-L1 regulation and offer potential advantages in molecular size, engineering flexibility, and future multispecific design.

**Supplementary Information:**

The online version contains supplementary material available at 10.1038/s41598-026-47884-x.

## Introduction

Immune checkpoint proteins are fundamental regulators of the immune system, maintaining immune homeostasis by modulating self-tolerance and preventing excessive immune activation^1^. However, tumors frequently exploit these inhibitory pathways to evade immune surveillance^2^. Among them, programmed death-ligand 1 (PD-L1) is widely recognized as a critical immune checkpoint regulator expressed on tumor cells and antigen-presenting cells^3,4^. PD-L1 interacts with its receptor programmed death-1 (PD-1) on activated T cells^5^, inhibiting T cell proliferation, cytokine secretion, and cytotoxic responses, which impairs antitumor immunity^6,7^.

Therapeutic targeting of PD-L1 using monoclonal antibodies—such as atezolizumab, durvalumab, avelumab, and cosibelimab—has demonstrated significant clinical benefit and established PD-L1 as a clinically validated immune checkpoint target^8–11^. Despite these advances, clinical responses are still limited to certain patient subsets^12,13^, likely due to the dynamic and heterogeneous nature of PD-L1 expression, along with variable co-expression patterns of other immune checkpoints and oncogenic factors in tumors^3,4^. Tumor subpopulations or metastatic lesions frequently express diverse combinations of inhibitory receptors (for example, PD-L1 with or without alternative checkpoints) and tumor-associated antigens, rendering single-target strategies insufficient for achieving durable disease control^14,15^. This biological complexity underscores the necessity for novel therapeutics capable of simultaneously modulating multiple immune checkpoints or tumor antigens^16,17^. However, most approved immunoglobulin G (IgG)-based monoclonal antibodies have been developed as single-target agents optimized to engage a single dominant antigen, which may limit their efficacy against tumors characterized by complex and heterogeneous antigenic landscapes^18,19^. Collectively, these biological and technical constraints highlight the critical need for alternative, modular antibody platforms that retain the validated mechanism of PD-L1 regulation while allowing straightforward reformatting into multispecific formats that incorporate additional immune checkpoints or tumor-associated molecules in a single multifunctional therapeutic entity. Consequently, these modular antibody platforms that support multispecific targeting are becoming more prominent in immuno-oncology.

Single-domain antibodies, also known as nanobodies or variable domains of heavy chain (VHHs), are derived from the variable domains of heavy-chain–only antibodies found in camelids and have emerged as a versatile and increasingly validated antibody format to address several of these challenges^20^. With a molecular weight of only ~ 15 kDa, VHHs are markedly smaller than conventional IgGs, enabling improved tissue penetration and access to sterically hindered epitopes^21^. Additionally, their simple architecture, combined with higher stability and solubility, supports cost-effective recombinant production and makes them highly amenable to engineering into multivalent, multispecific, or Fc-fusion formats. These attributes collectively facilitate efficient tissue targeting, economic manufacturing, and flexible integration of multiple effector functions^22^. In this context, VHH-based formats may also facilitate studies of PD-L1 regulation and offer potential advantages in terms of molecular size, engineering flexibility, and multispecific design. Several VHH-based therapeutics, including the FDA-approved caplacizumab, have advanced into clinical development, underscoring their translational potential^23^.

In this study, we isolated human PD-L1 (hPD-L1)-specific VHHs using phage display selection and reformatted them as Fc fusion proteins (VHH-Fc; K113.1-Fc and K113.2-Fc) to enhance their biophysical and functional properties. Both antibodies exhibited high-affinity binding to hPD-L1 and potent blockade of PD-1/PD-L1 interactions in vitro, thereby effectively relieving PD-1/PD-L1-mediated inhibitory signaling and restoring T cell activation. Notably, K113.2-Fc additionally promoted PD-L1 internalization and surface downregulation, correlating with enhanced T cell activation. This paired comparison between two closely related VHH-Fc antibodies with distinct functional behaviors provides a controlled framework for examining PD-L1 regulation and suggests the potential utility of VHH-based scaffolds as platforms for modular or multispecific checkpoint immunotherapeutics.

## Materials and methods

### Cell culture

MDA-MB-231 human triple-negative breast cancer (TNBC) cells (Korean Cell Bank, Seoul, Republic of Korea) and nuclear factor kappa-light-chain-enhancer of activated B cells (NF-κB)/Jurkat/green fluorescent protein (GFP™) transcriptional reporter cells (Jurkat-T-GFP; System Bioscience, Palo Alto, CA, USA) were cultured in Roswell Park Memorial Institute (RPMI) 1640 medium (RPMI1640; Gibco, Waltham, MA, USA) supplemented with 10% (v/v) fetal bovine serum (FBS; Gibco) and 1% (v/v) penicillin/streptomycin (Gibco) and maintained at 37 °C in a humidified 5% CO₂ incubator. Expi293F cells (Gibco) were cultured in Expi293 expression medium (Gibco) in a shaking incubator at 37 °C with 8% CO_2_.

### Isolation of hPD-L1-specific VHHs

hPD-L1-specific VHHs were isolated from our pre-established naïve alpaca VHH phage display library. The library used in this study was previously constructed and described^24^. Thus, no live animals were involved, and no additional blood collection or immunization was performed for the present work. The library was expanded in super broth (SB) medium (3% [w/v] tryptone, 2% [w/v] yeast extract, and 1% [w/v] 3-(N-morpholino) propanesulfonic acid [MOPS]; pH 7.0), aliquoted in 10% glycerol, and stored at − 80 °C. This library was subsequently rescued with VCSM13 helper phage and used for biopanning.

For antigen immobilization, 4 µg of hPD-L1 (Sino Biological, Beijing, China) was conjugated to M-270 Epoxy Dynabeads (Invitrogen, Waltham, MA, USA) according to the manufacturer’s instructions. Five rounds of biopanning were performed. In each round, hPD-L1 binding phages were retained on beads while unbound phages were removed by washing with phosphate-buffered saline (PBS) containing 0.1% (v/v) Tween-20 (PBST). Bound phages were eluted with 0.1 M glycine (pH 2.6) and neutralized with 1 M Tris-HCl (pH 8.9). Recovered phages were amplified using helper phage to enrich hPD-L1-specific binders. Additionally, 96 phage clones from the final round were randomly selected and screened for hPD-L1 reactivity using phage enzyme-linked immunosorbent assay (ELISA). Unique sequences were identified through DNA sequencing of the complementarity-determining regions (CDRs).

### Phage ELISA

The colonies selected from the five rounds of biopanning were inoculated into 1 mL of SB medium supplemented with 50 µg/mL carbenicillin in 96 deep-well plates (Axygen, Union City, CA, USA) and incubated at 37 °C for 6 h to allow phage production. After addition of 1 × 10^9^ plaque-forming units of VCSM13 helper phage and 70 µg/mL kanamycin, cultures were incubated overnight at 37 °C. The plates were then centrifuged at 3,000 g for 30 min to collect phage-containing supernatant. High-binding 96-well microplates (Corning, NY, USA) were coated with 0.1 µg/well of hPD-L1 protein (Sino Biological) overnight at 4 °C. After blocking with 3% (w/v) bovine serum albumin (BSA) in PBS, the plates were incubated with 100 µL of phage supernatant at 37 °C for 2 h, allowing the VHH-displaying phages to bind to their respective target antigens. The plates were washed thrice with 0.05% (v/v) PBST to remove unbound phages. Horseradish peroxidase (HRP)-conjugated anti–M13 antibody (1:5,000; Sino Biological) was added and incubated at 37 °C for 1 h to detect the bound phages. The plates were then washed three times with 0.05% (v/v) PBST, followed by the addition of the 3,3′,5,5′-tetramethylbenzidine substrate solution (Thermo Fisher Scientific, Waltham, MA, USA) for colorimetric detection. The enzymatic reaction was stopped by adding 2 N H_2_SO_4_, and absorbance was measured at 450 nm using a microplate reader (Synergy H1; BioTek, Winooski, VT, USA).

### Construction, expression, and purification of the selected VHH-Fc antibodies

Two VHH antibodies (K113.1 and K113.2) were subcloned into a pCEP4-derived mammalian expression vector encoding a wild-type human immunoglobulin G1 (IgG1) Fc domain to generate bivalent VHH-Fc fusion antibodies (K113.1-Fc and K113.2-Fc). Plasmids were transiently transfected into Expi293F cells (Gibco) using the Expi293 Expression System (Thermo Fisher Scientific) according to the manufacturer’s protocol. The secreted VHH-Fc antibodies were purified from culture supernatants using affinity chromatography with Protein A-Sepharose (Repligen, Waltham, MA, USA) and dialyzed in PBS.

### Surface plasmon resonance (SPR)

The binding kinetics of the selected VHH-Fc antibodies were analyzed through surface plasmon resonance (SPR) using an iMSPR-mini system (iCLUEBIO, Seongnam, Republic of Korea) with HBST buffer (10 mM HEPES, 150 mM NaCl, 0.005% [v/v] Tween-20, pH 7.4) as the running buffer. hPD-L1 (Sino Biological) was covalently immobilized onto a COOH–Au sensor chip (iCLUEBIO) via standard amine coupling. Serial dilutions of each VHH-Fc antibody (16, 32, 64, and 128 nM) were injected at a flow rate of 50 µL/min. The association and dissociation phases were set to 2 min and 5 min, respectively. After each cycle, the sensor surface was regenerated by injecting 10 mM glycine-HCl (pH 2.4) to remove residual bound antibodies. The equilibrium dissociation constant (K_D_) was determined using the iMSPR analysis software (TraceDrawer; iCLUEBIO).

To assess epitope overlap and competitive binding relationships among the antibodies, sequential binding/competition analyses were performed. Briefly, hPD-L1 (Sino Biological) was immobilized on a COOH-Au sensor chip (iCLUEBIO) using an amine coupling kit (iCLUEBIO) according to the manufacturer’s instructions. hPD-L1 was first saturated with 500 nM atezolizumab (Roche), followed by injection of either K113.1-Fc or K113.2-Fc (500 nM) to assess additional binding to the pre-occupied PD-L1 surface. In parallel competition experiments, hPD-L1 was initially saturated with K113.1-Fc (500 nM) followed by injection of K113.2-Fc (500 nM). In the reverse configuration, hPD-L1 was first saturated with K113.2-Fc and subsequently injected with K113.1-Fc (500 nM). All injections were performed at a flow rate of 50 µL/min at room temperature (RT). The resulting sensorgrams were analyzed using the iMSPR analysis software (TraceDrawer; iCLUEBIO).

### Flow cytometry

To assess the binding of the selected VHH-Fc antibodies to hPD-L1 on TNBC cells, MDA-MB-231 cells (1 × 10⁶) were fixed with 4% (w/v) paraformaldehyde (PFA), blocked with 1% (w/v) BSA in PBS, and incubated with 20 µg/mL of each VHH-Fc antibody at 4 °C for 1 h. Fixation was performed prior to antibody incubation to prevent antibody-induced internalization during the staining procedure. Because K113.2-Fc can promote PD-L1 endocytosis, surface-binding signals in live-cell assays may be underestimated. The fixation step stabilizes the cell surface and preserves intact PD-L1, allowing accurate measurement of antibody binding to the total surface-expressed antigen. After washing with PBS containing 1% (w/v) BSA, the cells were stained with fluorescein isothiocyanate (FITC)-conjugated anti–human IgG Fc antibody (1:100; Invitrogen) at 4 °C for 1 h. All samples were analyzed using a Guava easyCyte flow cytometer (Merck Millipore, Burlington, MA, USA), and data were processed with FlowJo software (TreeStar, Ashland, OR, USA).

To examine the effect of K113.2-Fc on the downregulation of hPD-L1 on the surface of TNBC cells, MDA-MB-231 cells (1 × 10^6^) were blocked with PBS containing 1% (w/v) BSA and treated with 20 µg/mL of K113.2-Fc or control VHH-Fc at 37 ℃ for 24 h. Subsequently, the cells were incubated with 20 µg/mL of anti–PD-L1 polyclonal antibody (Abcam, Cambridge, UK) at RT for 1 h. After washing with PBS containing 1% (w/v) BSA, the cells were stained with an FITC-labeled anti–mouse Fc antibody (1:200; Invitrogen) at RT for 1 h and analyzed as described above.

To exclude the possibility that the observed reduction in surface PD-L1 signal resulted from potential epitope masking effects, a fixation-based control experiment was performed using PFA-fixed MDA-MB-231 cells. Briefly, MDA-MB-231 cells (1 × 10⁶) were fixed with 4% (w/v) PFA and subsequently incubated in the presence or absence of 20 µg/mL K113.2-Fc in PBS containing 1% (*w*/*v*) BSA at RT for 1 h. After washing, the cells were stained with an anti–PD-L1 polyclonal antibody (Abcam) at RT for 1 h, followed by incubation with an FITC–labeled anti–mouse Fc secondary antibody (1:200; Invitrogen) at RT for 1 h, and analyzed as described above.

### Protein–protein interaction inhibition assay

The inhibitory effect of hPD-L1-specific VHH-Fc antibodies against PD-1/PD-L1 interactions was assessed using ELISA. Fc-tagged hPD-1 (10 nM; Sino Biological) was coated onto a high-binding 96-well microplate (Corning) and incubated at RT for 2 h. The wells were blocked with 3% (w/v) BSA in PBST for 2 h at RT. In parallel, His-tagged hPD-L1 (20 nM; Sino Biological) was preincubated with either K113.1-Fc, K113.2-Fc, or atezolizumab at the indicated concentrations (0, 10, 50, and 250 nM) at RT for 2 h. The mixtures were then added to the hPD-1-coated wells and incubated at RT for 1 h. The wells were washed an additional three times with 0.05% (v/v) PBST, HRP-conjugated anti–His antibody (1:5,000; R&D Systems) was added and incubated at RT for 1 h. After washing thrice with 0.05% (v/v) PBST, chemiluminescent signals were measured using the SuperSignal ELISA Pico Chemiluminescent Substrate (Thermo Fisher Scientific) and measured at 425 nm using a microplate reader (Synergy H1; BioTek). Absorbance values were normalized to those of the control wells without antibody treatment.

### Measurement of antibody internalization

Antibody internalization of the selected VHH-Fc antibodies in MDA-MB-231 cells was assessed using FabFluor-pH Red antibody labeling reagent (Sartorius, Göttingen, Germany) according to the manufacturer’s instructions. To allow cell adhesion, MDA-MB-231 cells were seeded at 5 × 10^3^ cells per well in a 96-well culture plate (Falcon, Corning) and incubated overnight. K113.1-Fc or K113.2-Fc antibodies were labeled with FabFluor-pH Red reagent at a 1:3 molar ratio in PBS and incubated at RT for 15 min. The labeled antibodies were added to the culture medium at a final concentration of 4 µg/mL. For live-cell imaging, the plates were transferred to the IncuCyte SX1 Live-Cell Analysis System (Sartorius), and images were obtained with 10× objective for 12 h. The fluorescence intensity in each well was quantified using the IncuCyte analysis software (Sartorius).

### T cell activation assay

To evaluate the ability of the selected VHH-Fc antibodies to restore T cell activation suppressed by PD-L1-expressing MDA-MB-231 cells, 3 × 10^4^ Jurkat-T-GFP cells were seeded into each well of a 96-well culture plate (Falcon) that was either uncoated or coated with 1.5 × 10^5^ cells of MDA-MB-231. The Jurkat-T-GFP cells were stimulated with 1 µg/mL anti–CD3ε antibody in the presence or absence of 20 µg/mL of the selected VHH-Fc antibodies or atezolizumab at 37 °C for 2 h. Then, live-cell images were obtained using the IncuCyte SX1 Live-Cell Analysis System (Sartorius) equipped with a 10× objective for 24 h. Furthermore, GFP fluorescence intensity was quantified using the IncuCyte analysis software (Sartorius).

### Statistical analyses

All statistical analyses were performed using GraphPad Prism (GraphPad Software Inc., San Diego, CA, USA). A Dunnett’s multiple-comparisons test was used to compare each treatment group with the MOCK control, and *p*-values of < 0.05 were considered statistically significant (*p < 0.05, **p < 0.01, ***p < 0.001, and ****p < 0.0001). Data are presented as the mean ± standard deviation (SD) from independent experiments, as indicated in the figure legends. For experiments involving more than two groups, one-way ANOVA was used as specified in the corresponding Results and figure legends.

## Results

### Selection of hPD-L1-specific VHHs and reformatting into VHH-Fc antibodies

**Fig. 1:**
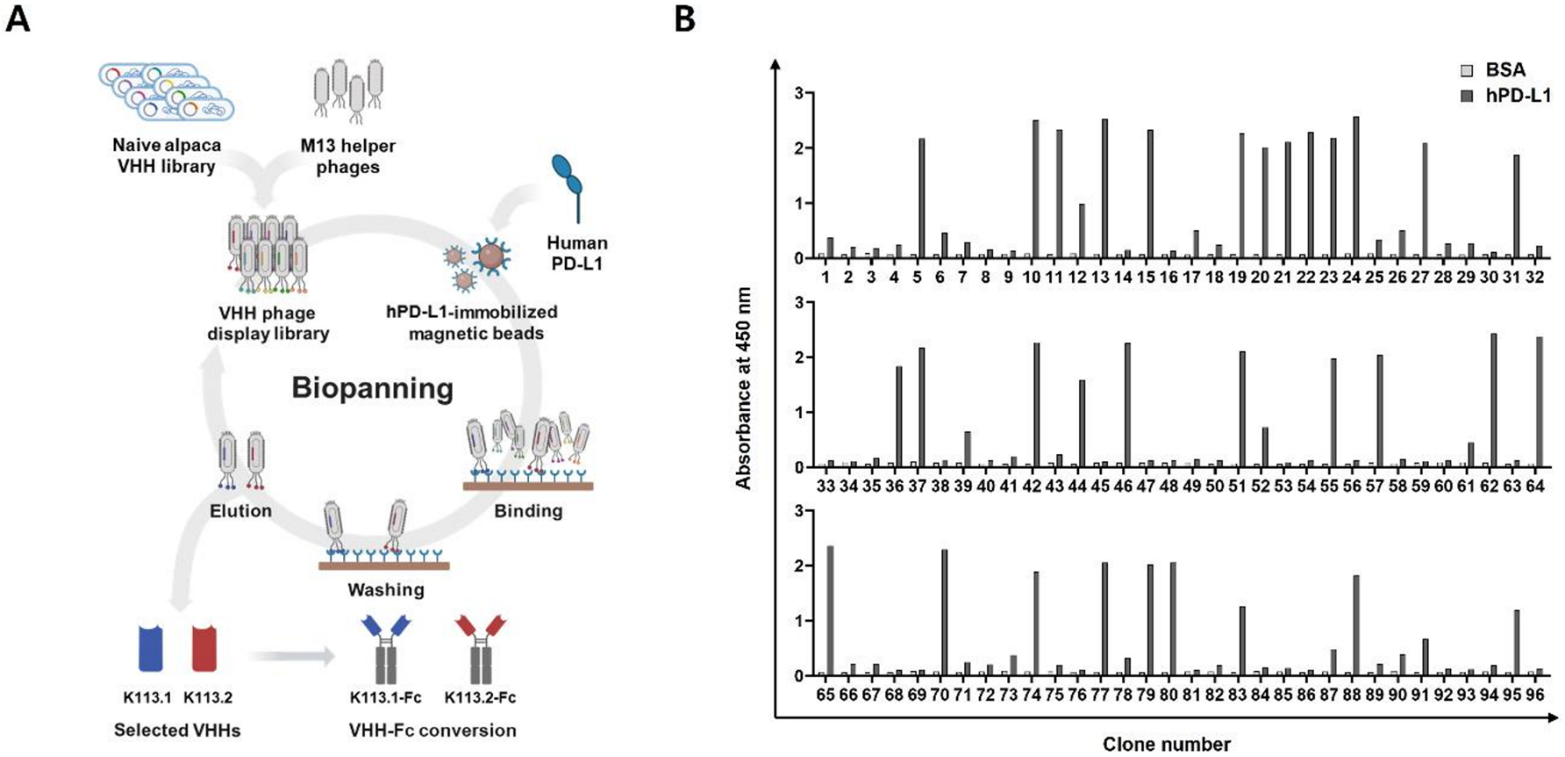
Selection of hPD-L1-specific VHHs and reformatting into VHH-Fc antibodies.

To isolate VHHs specific to hPD-L1, we performed biopanning using a pre-existing naïve alpaca VHH phage display library. This library was previously constructed from lymphocytes collected from 20 non-immunized alpacas, as detailed in our previous study^24^. The library comprises approximately 7.16 × 10^10^ independent clones, providing a highly diverse repertoire for isolating high-affinity binders. hPD-L1 immobilized on magnetic beads served as the target antigen. Five rounds of biopanning were conducted on the phage library, using progressively stringent washing conditions to enrich phages displaying high-affinity hPD-L1-specific VHHs (Fig. 1A). Following the final biopanning round, 96 individual phage clones were randomly selected from the output pool and screened using phage ELISA. Among these clones, multiple candidates exhibited strong and specific binding to hPD-L1, whereas no binding was observed against BSA, which was used as a negative control (Fig. 1B). From these positive binders, two unique clones, K113.1 and K113.2, were selected based on their strong binding signals and distinct CDR sequences. DNA sequencing confirmed that these clones contained unique CDR regions. These VHH clones were subsequently reformatted into bivalent Fc-fusion antibodies by subcloning the VHH sequences into a pCEP4-derived mammalian expression vector encoding a wild-type human IgG1 Fc domain. The resulting antibodies were designated as K113.1-Fc and K113.2-Fc.

### Biophysicochemical characterization of hPD-L1-specific VHH-Fc antibodies

**Fig. 2:**
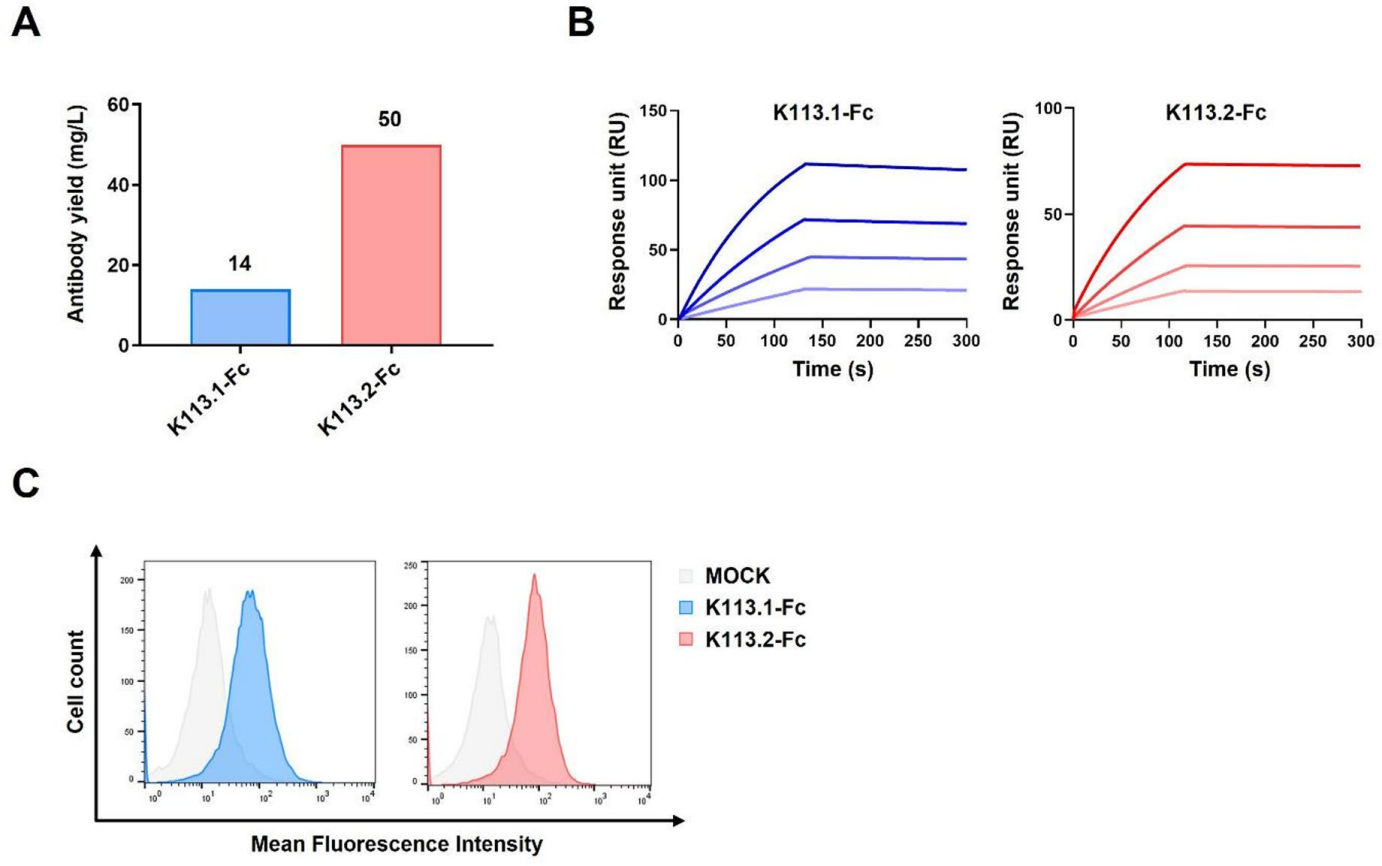
Biophysicochemical characterization of the selected VHH-Fc antibodies.

To assess the biophysicochemical properties of the selected hPD-L1-specific VHH-Fc antibodies, K113.1-Fc and K113.2-Fc were transiently expressed in mammalian cells and purified from the culture supernatants using Protein A-Sepharose affinity chromatography, followed by buffer exchange into PBS. The final production yields were approximately 14 mg/L for K113.1-Fc and 50 mg/L for K113.2-Fc, indicating higher expression efficiency of K113.2-Fc (Fig. 2A).

Furthermore, SPR analysis was performed to determine the binding kinetics of the selected VHH-Fc antibodies. After immobilizing hPD-L1 onto a sensor chip via standard amine coupling, antigen–antibody binding kinetics were measured across serially diluted concentrations of each antibody. Both antibodies exhibited high-affinity binding to hPD-L1, with K_D_ values of 3.36 and 2.09 nM for K113.1-Fc and K113.2-Fc, respectively (Fig. 2B; Table 1).

**Table 1:** Binding affinities of the selected VHH-Fc antibodies against hPD-L1.

Antibody (VHH-Fc)	K_D_ (nM)	K_a_ (M^− 1^ s^− 1^)	K_d_ (s^− 1^)
K113.1-Fc	3.36	6.92 × 10^4^	2.33 × 10^− 4^
K113.2-Fc	2.09	3.26 × 10^4^	6.82 × 10^− 5^

K_D_, equilibrium dissociation constant; K_a_, association constant; and K_d_, dissociation constant.

Flow cytometry analysis was then conducted to confirm the ability of the selected antibodies to recognize PD-L1 expressed on the cell surface. Both K113.1-Fc and K113.2-Fc efficiently bound to PD-L1 expressed on MDA-MB-231 cells, whereas negligible binding was observed in the MOCK control (Fig. 2C). Collectively, these findings demonstrate that both VHH-Fc antibodies possess robust biophysical integrity and high-affinity antigen binding. Although K113.2-Fc displayed a higher production yield, both antibodies exhibited comparable biophysical quality, supporting their use in subsequent functional characterization.

### Elucidation of the distinct action mechanism of hPD-L1-specific VHH-Fc antibodies: receptor blockade and PD-L1 downregulation via antibody internalization

**Fig. 3:**
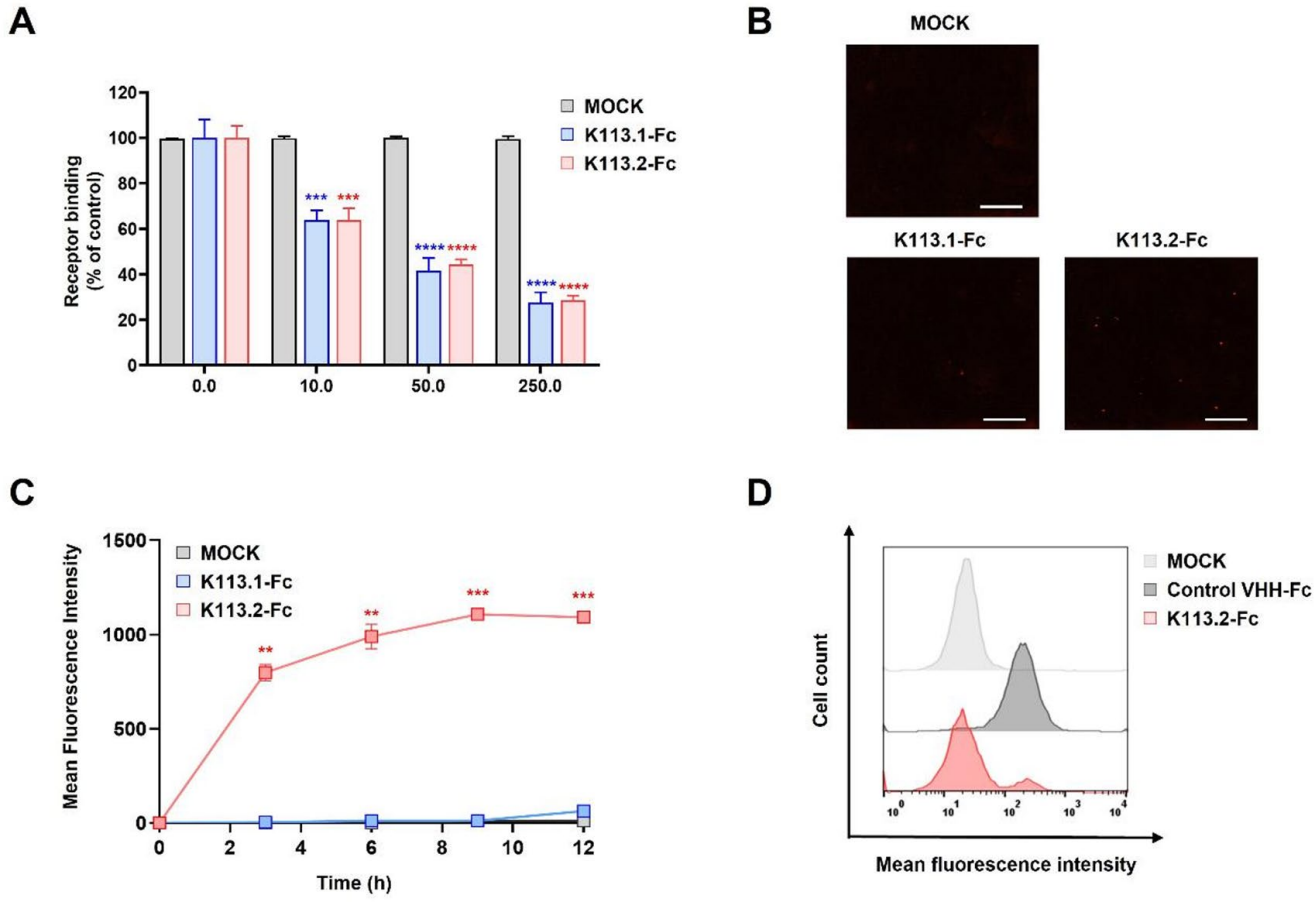
Functional characterization of the selected VHH-Fc antibodies through receptor–ligand blockade, internalization, and surface downregulation.

To investigate the functional activity of the selected VHH-Fc antibodies, we first assessed their ability to inhibit the interaction between PD-L1 and its receptor PD-1 using an ELISA-based receptor–ligand inhibition assay. To ensure quantitative assessment within a reliable detection range, we first generated a PD-1/PD-L1 binding curve by varying the concentrations of recombinant proteins and selected assay conditions within the linear range for subsequent inhibition experiments. In this context, the primary objective was to determine whether the two antibodies we developed, K113.1-Fc and K113.2-Fc, possess differentiated functional characteristics. Under these optimized conditions, both antibodies showed clear concentration-dependent inhibition, confirming their functional blockade activity, whereas the MOCK control showed negligible inhibition across all tested concentrations (Fig. 3A). As a clinically validated positive control, atezolizumab (FDA-approved anti–PD-L1 IgG1) was tested in parallel and achieved a higher level of inhibition than K113.2-Fc, approaching maximal inhibition under these assay conditions (Supplementary Fig. S1). This result confirms that the assay system is capable of detecting near-complete blockade and providing a clinically relevant reference for interpreting the inhibitory activity of our VHH-Fc antibodies. These results suggest that the selected antibodies may effectively interfere with the PD-1/PD-L1 interaction and can potentially exert their receptor-blocking activity for attenuating this immunosuppressive signaling axis.

Consistent with these findings, SPR-based competition analysis showed that when PD-L1 was first saturated with atezolizumab, subsequent injection of K113.1-Fc or K113.2-Fc still resulted in measurable binding signals (Supplementary Fig. S2A,B). These results suggest that K113.1-Fc and K113.2-Fc binding sites are not fully overlapping with those of atezolizumab, indicating distinct or partially overlapping binding regions.

We next examined whether the selected antibodies could mediate internalization of cell surface PD-L1. Live-cell imaging revealed a pronounced uptake of K113.2-Fc into MDA-MB-231 cells over a 12 h period, whereas K113.1-Fc showed weaker internalization and the control antibody showed minimal uptake (Fig. 3B). Quantitative analysis confirmed a time-dependent increase in intracellular fluorescence intensity for K113.2-Fc, which remained consistently higher than that for K113.1-Fc (Fig. 3C). These results indicate that K113.2-Fc induce PD-L1 internalization, potentially leading to decreased PD-L1 levels on the tumor cell surface.

Since K113.2-Fc exhibited pronounced internalization in MDA-MB-231 cells expressing PD-L1, we next assessed whether this internalization led to a reduction in cell surface PD-L1 expression. MDA-MB-231 cells were treated with control VHH-Fc or K113.2-Fc for 24 h, and cell surface PD-L1 levels were subsequently analyzed using flow cytometry. Compared to control VHH-Fc, K113.2-Fc treatment markedly reduced the surface expression of PD-L1 on MDA-MB-231 cells, suggesting that K113.2-Fc may promote PD-L1 downregulation (Fig. 3D).

To address the possibility that the reduced PD-L1 signal resulted from competitive epitope masking rather than true internalization, we performed a fixation-based control experiment. MDA-MB-231 cells were fixed prior to antibody treatment to prevent endocytosis and membrane trafficking. Under these conditions, K113.2-Fc did not reduce PD-L1 staining, and the signal remained comparable to that of untreated controls, indicating that the decreased PD-L1 signal observed in live cells reflects true internalization rather than an artifact of epitope masking (Supplementary Fig. S3).

Beyond these observations, the structural relationship between the two VHH-Fc antibodies was further investigated. SPR analysis revealed order-dependent competition, suggesting a hierarchical epitope relationship where the K113.1-Fc binding site is contained within the broader boundary recognized by K113.2-Fc (Supplementary Fig. S4A,B). This expanded epitope engagement by K113.2-Fc likely provides the structural basis for triggering efficient PD-L1 internalization—a feature absent in K113.1-Fc despite their shared core binding region.

Taken together, these findings demonstrate that both K113.1-Fc and K113.2-Fc have the potential to inhibit the PD-1/PD-L1 interaction, thereby possibly attenuating immune checkpoint signaling. Notably, K113.2-Fc additionally appears to induce PD-L1 internalization and reduce surface expression levels, suggesting a distinct functional profile consistent with a dual mechanism of action relative to K113.1-Fc.

### Restoration of T cell activation by hPD-L1-specific VHH-Fc antibodies

To determine whether the hPD-L1-specific VHH-Fc antibodies could restore T cell function, we employed a GFP-based reporter assay using the Jurkat-T-GFP cell system. The Jurkat-T-GFP cells, a Jurkat-derived NF-κB reporter cell line, enable the direct visualization of T cell activation through GFP expression. Jurkat-T-GFP reporter cells stimulated with anti–CD3ε antibody, a well-known activator of the T cell receptor complex, were used as a positive control and defined as 100% activation. In this co-culture system, PD-L1-expressing MDA-MB-231 cells markedly suppressed T cell activation to approximately 40% of the control level, consistent with PD-L1-mediated immune inhibition.

**Fig. 4:**
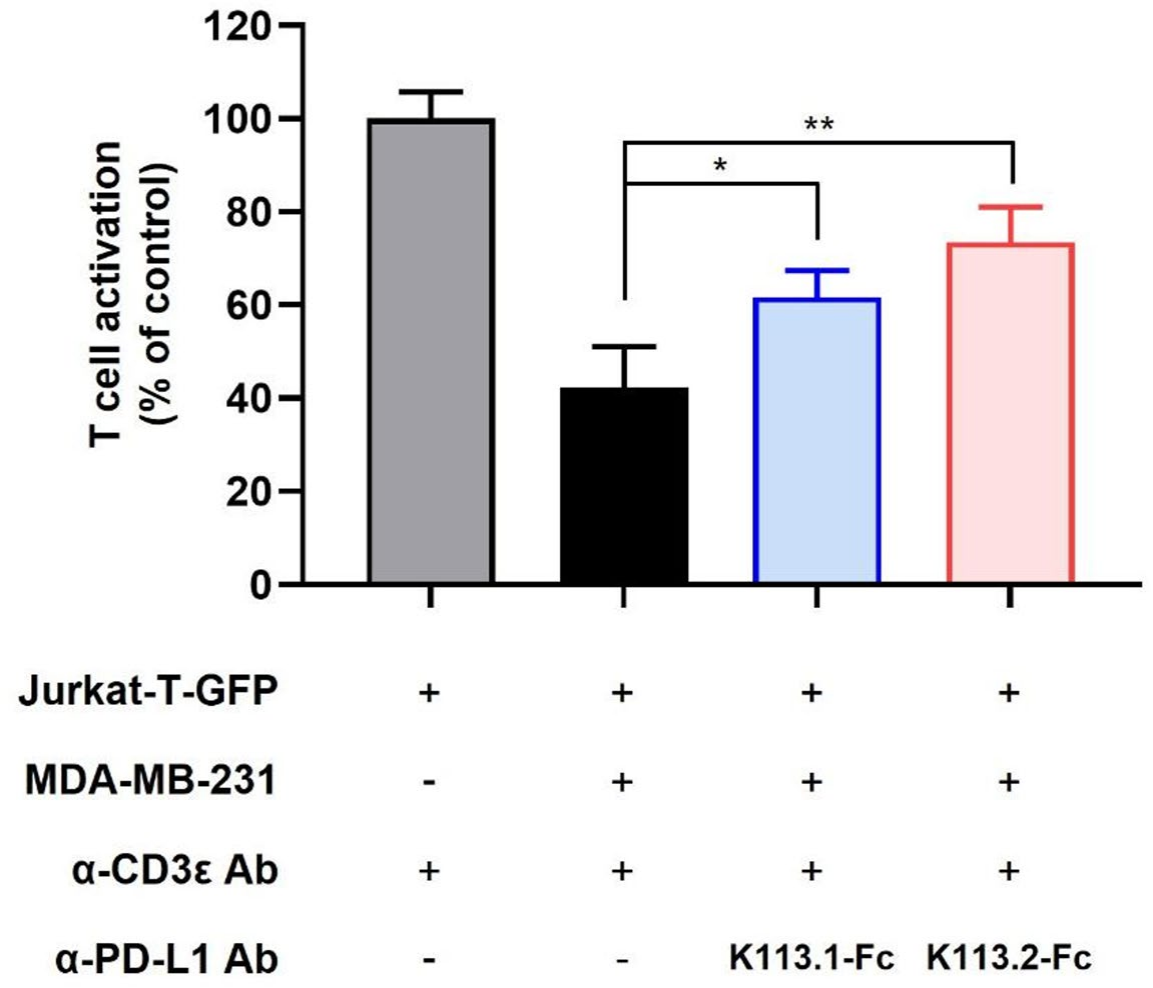
Functional effect of the selected VHH-Fc antibodies on T cell activation.

Consistent with the observed molecular activities, treatment with VHH-Fc antibodies alleviated this suppression in a clone-dependent manner. K113.1-Fc partially restored activation to ~ 60% of control levels, whereas K113.2-Fc, which additionally mediates PD-L1 internalization and downregulation, achieved a stronger recovery of ~ 80% (Fig. 4). The observed effects were statistically significant (**p < 0.01). Error bars represent mean ± SD from three independent experiments, and statistical significance was determined by comparison between the indicated antibody-treated groups using one-way ANOVA with Dunnett’s multiple-comparisons test. Collectively, these findings suggest that the dual activity of K113.2-Fc—blocking PD-1/PD-L1 interaction and lowering cell-surface PD-L1 levels—may enhance restoration of T cell activation.

In addition, a comparative experiment was performed using the clinically validated anti–PD-L1 antibody atezolizumab under the same co-culture conditions. Both atezolizumab and K113.2-Fc induced comparable levels of T cell activation, whereas K113.1-Fc exhibited relatively lower activity. These results further support the functional activity of K113.2-Fc and provide a clinically relevant reference for interpreting its activity (Supplementary Fig. S5).

## Discussion

PD-L1 is a key immune checkpoint molecule that regulates T cell activity through engagement with its inhibitory receptor PD-1^25^. Its aberrant expression across multiple tumor types is associated with impaired immune function, unfavorable prognosis, and therapeutic resistance^26^. Although monoclonal antibodies targeting PD-1 or PD-L1 have transformed cancer immunotherapy, limited response rates and resistance in many patients remain major clinical challenges^27^. These therapeutic constraints may stem from tumor-intrinsic heterogeneity, the dynamic regulation of PD-L1 expression, and the single-target constraints of conventional IgG formats^28,29^. These complexities underscore the need for therapeutic approaches that can modulate multiple immune checkpoints or tumor-associated antigens within a single framework. Consequently, alternative antibody scaffolds that maintain effective PD-1/PD-L1 blockade and provide greater flexibility for multispecific or functionally enhanced configurations have garnered increasing interest^30^. A deeper understanding of PD-L1 regulation may provide useful insights for designing next-generation immune checkpoint therapeutics.

In this study, we developed and characterized two PD-L1-specific VHH-Fc antibodies (K113.1-Fc and K113.2-Fc) and investigated their potential mechanisms of action. Both antibodies exhibited high affinity and specificity for hPD-L1 and blocked PD-1/PD-L1 interactions, confirming their capacity to neutralize PD-L1-mediated immune suppression. Functional assays further demonstrated that these antibodies could restore T cell activation suppressed by PD-L1-expressing tumor cells, suggesting that VHH-Fc antibodies may act as compact and modular scaffolds for immune checkpoint modulation.

PD-L1 internalization has been reported for several previously described antibodies, including clinically used agents, indicating that this phenomenon is not unique to the antibodies described in this study. Although the two antibodies shared a receptor–ligand blocking mechanism, they displayed distinct functional characteristics. K113.1-Fc primarily acted through PD-1/PD-L1 blockade, whereas K113.2-Fc additionally induced PD-L1 internalization and a reduction in cell surface PD-L1 levels. This internalization-driven reduction in surface PD-L1 availability may provide a secondary mechanism through which K113.2-Fc could potentially contribute to checkpoint modulation beyond simple receptor–ligand competitive blockade. These mechanistic differences were reflected in T cell activation assays. Both antibodies alleviated PD-L1-dependent suppression to a measurable extent, but K113.2-Fc resulted in a more robust recovery of activation signals than K113.1-Fc. This enhanced effect suggests that the additional mechanism associated with K113.2-Fc, including antibody-induced PD-L1 downregulation, contributes to improved restoration of immune activity. Our competition analysis further suggests that this functional divergence may stem from a hierarchical epitope relationship, where K113.2-Fc appears to recognize a broader boundary that encompasses the K113.1-Fc binding site (Supplementary Fig. S4A,B). The functional potency of K113.2-Fc was further supported by its robust activity in the T cell co-culture assay, which was evaluated alongside the clinically validated antibody atezolizumab as a reference (Supplementary Fig. S5). This suggests that the dual mechanism of K113.2-Fc—combining blockade with surface downregulation—may provide an alternative and effective strategy for modulating PD-L1 activity, especially in cellular environments where surface PD-L1 availability plays a significant role. Decreasing surface PD-L1 may reduce engagement of PD-1 and other co-regulatory partners such as CD80, thereby influencing various immunoregulatory pathways. These effects are especially important in tumors with high PD-L1 turnover or compensatory upregulation, where ongoing checkpoint inhibition may require modulation of both PD-L1 activity and its surface expression levels^31,32^. In addition, post-translational modifications of PD-L1, such as glycosylation, may influence its trafficking, stability, and internalization dynamics, and warrant further mechanistic investigation. However, the observed enhancement in T cell activation remains to be further clarified, including whether it primarily results from direct modulation of T cell signaling or from reduced PD-L1 availability. In this context, future studies analyzing PD-L1 trafficking, intracellular degradation, and recycling following antibody engagement will be valuable for elucidating the biological significance of this internalization process^33^.

In the PD-1/PD-L1 blockade assay, both VHH-Fc antibodies showed a maximal inhibition plateau of approximately 70%. Because the assay was performed within the linear detection range, and the control antibody atezolizumab achieved near-complete inhibition under the same conditions, this plateau is unlikely to reflect a technical limitation of the ELISA system. Instead, this observation may result from differences in the binding sites of the VHH-Fc antibodies relative to those of atezolizumab, which may lead to incomplete steric blockade of the PD-1/PD-L1 interaction. Consistent with this interpretation, SPR competition analysis demonstrated that K113.1-Fc and K113.2-Fc retained measurable binding after PD-L1 saturation with atezolizumab, suggesting that their binding sites are not fully overlapping with that of atezolizumab. This non-identical epitope relationship may account for the partial inhibition observed in the ELISA assay. Nevertheless, the restoration of T cell activity observed in our co-culture experiments suggests that this level of blockade, when combined with the distinct properties of these VHH-Fc antibodies, may be functionally sufficient to alleviate PD-L1-mediated immune suppression.

The modular architecture of the VHH-Fc format also offers substantial opportunities for further engineering and functional enhancement^34^. Unlike conventional IgG scaffolds, the compact size, stability, and solubility of VHH domains enable efficient assembly of multivalent or multispecific configurations without compromising manufacturability^21^. These structural features facilitate the straightforward combination of PD-L1 targeting with additional immune regulators (e.g., TIGIT, LAG-3, Siglec-15), providing a practical strategy to address pathway redundancy and the heterogeneous inhibitory landscapes that underlie resistance to current immune checkpoint therapies^35–37^. In addition, antibodies with efficient internalization properties, such as K113.2-Fc, could potentially serve as scaffolds for targeted delivery systems—including antibody–drug conjugates or immunocytokine platforms—directed toward PD-L1–expressing tumors^38^. Furthermore, these antibodies may be applied as molecular tools for studying PD-L1 trafficking, turnover, and degradation, thereby contributing to a more comprehensive understanding of its biology^39^.

Taken together, this study presents two hPD-L1-specific VHH-Fc antibodies with distinct mechanisms of action. Both K113.1-Fc and K113.2-Fc disrupted PD-1/PD-L1 interactions and alleviated PD-L1-mediated immune suppression in vitro, while K113.2-Fc uniquely induced PD-L1 internalization and surface downregulation. In this context, the VHH-based format may facilitate studies of PD-L1 regulation and offer potential advantages in terms of molecular size, engineering flexibility, and multispecific design. Importantly, the present findings are limited to in vitro systems, and further functional validation, including in vivo or organoid-based studies, will be necessary to determine their biological and potential therapeutic relevance. Functional validation using primary human T cells would also strengthen the physiological relevance of these findings, and such studies will be pursued in future work. This dual mechanism may provide an alternative strategy for modulating PD-L1 activity under immunosuppressive conditions. These findings offer initial insights into how VHH-Fc antibodies may influence PD-L1 regulation and establish a foundation for future in vivo and translational studies. Overall, the results suggest that hPD-L1-specific VHH-Fc antibodies may serve as modular platforms for investigating PD-L1 regulation and for developing next-generation immune checkpoint therapeutics.

## Supplementary Information

Below is the link to the electronic supplementary material.

Supplementary Material 1

## Data Availability

The data supporting the findings of this study are available from the corresponding author upon reasonable request.
