# Supplementary material for: Differential T cell reactivation by two PD-L1 nanobodies through blockade alone or blockade with internalization
Source: Sci Rep. 2026 Apr 18;16:18095. doi: 10.1038/s41598-026-47884-x (PMC13254401; doi:10.1038/s41598-026-47884-x)
Supplement: Supplementary file 1 — Supplementary Material 1 [file 41598_2026_47884_MOESM1_ESM.pdf]

Discovery of Two PD-L1 Nanobodies with Distinct Mechanisms—Blockade versus Blockade/Internalization for T Cell Reactivation

Authors: Ji Hyun Lee[1,†], Su Yeon Cho[1,†], Hee Eon Lee[1], and Sukmook Lee[1,2,3,4,*]

Affiliation:

[1]Department of Biopharmaceutical Chemistry, Kookmin University, Seoul 02707, Republic of Korea

[2]Department of Applied Chemistry, Kookmin University, Seoul 02707, Republic of Korea

[3]Antibody Research Institute, Kookmin University, Seoul 02707, Republic of Korea

[4]Department of Neurosurgery, School of Medicine, Stanford University, Palo Alto, CA 94304, USA

†These authors contributed equally to this work.

Running title: PD-L1 Nanobodies for T Cell Activation

***Corresponding Author's Information**:

Sukmook Lee, Tel.: +82-2-910-6763; Fax: +82-2-910-4115; E-mail: lees2018@kookmin.ac.kr

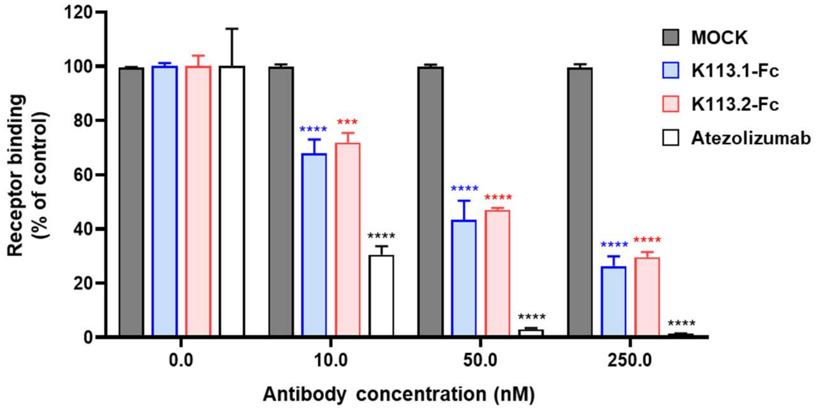

Supplementary Figure 1. ELISA-based receptor–ligand blockade of PD-1/PD-L1 by the selected VHH-Fc antibodies and atezolizumab. ELISA-based receptor–ligand inhibition assays demonstrating the dose-dependent reduction in PD-1/PD-L1 binding by K113.1-Fc (blue) and K113.2-Fc (red), with atezolizumab (white), an FDA-approved anti–PD-L1 monoclonal antibody, included as a positive control to validate assay sensitivity. MOCK control (gray) served as the untreated binding control. The percentage of PD-1/PD-L1 receptor binding was calculated as follows: Receptor binding (% of control) = $[(OD^{Sample} - OD^{Background}) / (OD^{MOCK} - OD^{Background})] \times 100$. Data are presented as mean ± standard deviation (SD) of three independent experiments performed in triplicate. Statistical significance was determined using one-way ANOVA with Dunnett's multiple-comparisons test, with comparisons made against MOCK control (gray). ***$p < 0.001$, ****$p < 0.0001$.

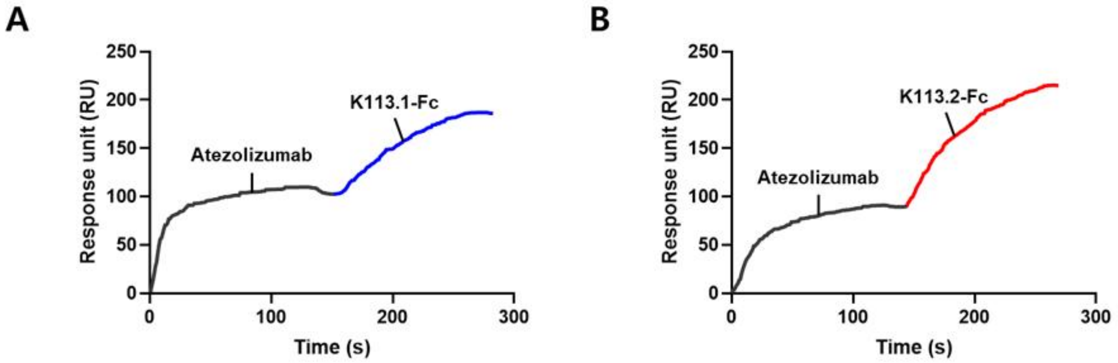

Supplementary Figure 2. Comparison of the binding sites of the selected antibodies on PD-L1 with those of atezolizumab. SPR sensorgrams showing sequential binding of atezolizumab and K113.1-Fc, or K113.2-Fc to immobilized hPD-L1. (A) SPR sensorgrams illustrating additional binding of K113.1-Fc (blue) to hPD-L1 presaturated with atezolizumab (black). (B) SPR sensorgrams showing additional binding of K113.2-Fc (red) to hPD-L1 presaturated with atezolizumab (black).

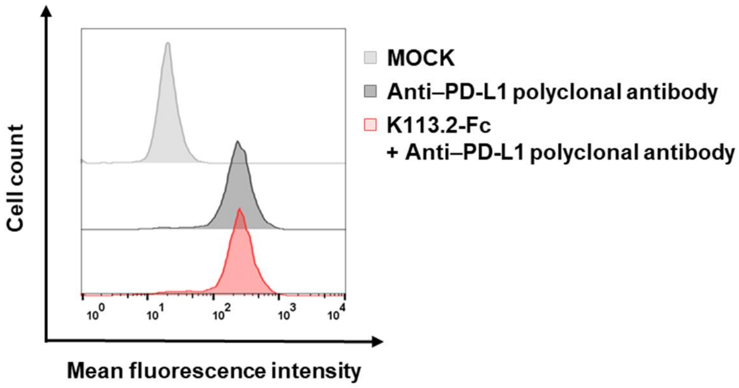

Supplementary Figure 3. Binding of the anti–PD-L1 polyclonal antibody to MDA-MB-231 cells. Flow cytometry analysis of the effect of K113.2-Fc on the binding of an anti–PD-L1 polyclonal antibody to MDA-MB-231 cells. Paraformaldehyde-fixed MDA-MB-231 cells were either treated with the anti–PD-L1 polyclonal antibody in the absence (dark gray) or presence (red) of K113.2-Fc. Cells treated with the secondary antibody alone (light gray) served as the MOCK control (negative control). The overlapping fluorescence intensity between the K113.2-Fc-treated group (red), and the polyclonal antibody-only group (dark gray) indicates that K113.2-Fc does not competitively inhibit or displace the binding of the polyclonal antibody.

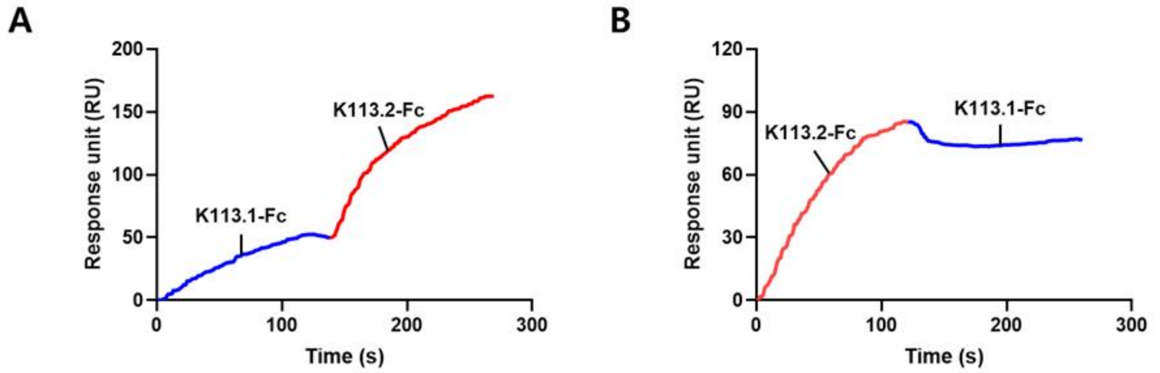

Supplementary Figure 4. Competition analysis of PD-L1 binding sites of the selected VHH-Fc antibodies. SPR sensorgrams showing sequential binding of K113.1-Fc or K113.2-Fc to immobilized hPD-L1. (A) SPR sensorgrams showing sequential injection of K113.2-Fc (red) onto hPD-L1 presaturated with K113.1-Fc (blue). (B) SPR sensorgrams showing sequential injection of K113.1-Fc (blue) onto hPD-L1 presaturated with K113.2-Fc (red).

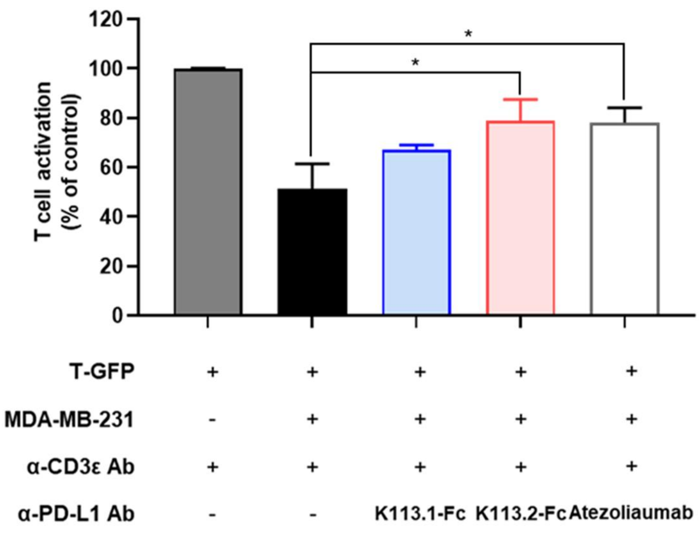

Supplementary Figure 5. Functional characterization of T cell activation by the selected VHH-Fc antibodies and atezolizumab. T cell activation was evaluated by measuring GFP fluorescence in Jurkat-T-GFP reporter cells. Jurkat-T-GFP cells stimulated with an anti–CD3ε antibody alone were used as the positive activation control (gray; 100% activation). Jurkat-T-GFP cells were co-cultured with PD-L1-expressing MDA-MB-231 cells in the absence of antibody (black) or in the presence of K113.1-Fc (blue), K113.2-Fc (red), or atezolizumab (white). GFP fluorescence intensity served as a quantitative readout of reporter cell activation. Data are shown as mean ± SD from three independent experiments performed in triplicate. Statistical significance was assessed using one-way ANOVA with Dunnett's multiple-comparisons test, with comparisons made against the untreated control group (black). *$p < 0.05$.
